# Sequential implementation of DSC-MR perfusion and dynamic [^18^F]FET PET allows efficient differentiation of glioma progression from treatment-related changes

**DOI:** 10.1007/s00259-020-05114-0

**Published:** 2020-11-26

**Authors:** Eike Steidl, Karl-Josef Langen, Sarah Abu Hmeidan, Nenad Polomac, Christian P. Filss, Norbert Galldiks, Philipp Lohmann, Fee Keil, Katharina Filipski, Felix M. Mottaghy, Nadim Jon Shah, Joachim P. Steinbach, Elke Hattingen, Gabriele D. Maurer

**Affiliations:** 1 Institute of Neuroradiology, University Hospital, Goethe University Frankfurt am Main, Schleusenweg 2-16, Frankfurt am Main, 60528 Germany; 2 University Cancer Center Frankfurt (UCT), University Hospital, Goethe University Frankfurt am Main, Frankfurt am Main, Germany; 3 German Cancer Consortium (DKTK), Partner Site Frankfurt/Mainz, and German Cancer Research Center (DKFZ), Heidelberg, Germany; 4 Inst. of Neuroscience and Medicine, Cognitive Neuroscience (INM-3), Research Center Juelich, Juelich, Germany; 5 Inst. of Neuroscience and Medicine, Medical Imaging Physics (INM-4), Research Center Juelich, Juelich, Germany; 6 Dept. of Nuclear Medicine, University Hospital RWTH Aachen, Aachen, Germany; 7 Center for Integrated Oncology (CIO), Universities of Aachen, Bonn, Cologne, and Duesseldorf, Aachen, Germany; 8 Dept. of Neurology, Faculty of Medicine and University Hospital Cologne, University of Cologne, Cologne, Germany; 9 Institute of Neurology (Edinger Institute), University Hospital, Goethe University Frankfurt am Main, Frankfurt am Main, Germany; 10 Dept. of Radiology and Nuclear Medicine, Maastricht University Medical Center (MUMC+), Maastricht, The Netherlands; 11 Inst. of Neuroscience and Medicine, Molecular Neuroscience and Neuroimaging (INM-11), JARA, Research Center Juelich, Juelich, Germany; 12 Dept. of Neurology, University Hospital RWTH Aachen, Aachen, Germany; 13 Dr. Senckenberg Institute of Neurooncology, University Hospital, Goethe University Frankfurt am Main, Frankfurt am Main, Germany; 14 Center for Integrated Oncology (CIO), Universities of Aachen, Bonn, Cologne, and Duesseldorf, Cologne, Germany; 15 JARA - BRAIN - Translational Medicine, Aachen, Germany; 16 Department of Stereotaxy and Functional Neurosurgery, Faculty of Medicine and University Hospital Cologne, University of Cologne, Cologne, Germany

**Keywords:** Glioma, PWI, [^18^F]FET PET, Pseudoprogression, Isocitrate dehydrogenase

## Abstract

**Purpose:**

Perfusion-weighted MRI (PWI) and O-(2-[^18^F]fluoroethyl-)-**l**-tyrosine ([^18^F]FET) PET are both applied to discriminate tumor progression (TP) from treatment-related changes (TRC) in patients with suspected recurrent glioma. While the combination of both methods has been reported to improve the diagnostic accuracy, the performance of a sequential implementation has not been further investigated. Therefore, we retrospectively analyzed the diagnostic value of consecutive PWI and [^18^F]FET PET.

**Methods:**

We evaluated 104 patients with WHO grade II–IV glioma and suspected TP on conventional MRI using PWI and dynamic [^18^F]FET PET. Leakage corrected maximum relative cerebral blood volumes (rCBV_max_) were obtained from dynamic susceptibility contrast PWI. Furthermore, we calculated static (i.e., maximum tumor to brain ratios; TBR_max_) and dynamic [^18^F]FET PET parameters (i.e., Slope). Definitive diagnoses were based on histopathology (*n* = 42) or clinico-radiological follow-up (*n* = 62). The diagnostic performance of PWI and [^18^F]FET PET parameters to differentiate TP from TRC was evaluated by analyzing receiver operating characteristic and area under the curve (AUC).

**Results:**

Across all patients, the differentiation of TP from TRC using rCBV_max_ or [^18^F]FET PET parameters was moderate (AUC = 0.69–0.75; *p* < 0.01). A rCBV_max_ cutoff > 2.85 had a positive predictive value for TP of 100%, enabling a correct TP diagnosis in 44 patients. In the remaining 60 patients, combined static and dynamic [^18^F]FET PET parameters (TBR_max_, Slope) correctly discriminated TP and TRC in a significant 78% of patients, increasing the overall accuracy to 87%. A subgroup analysis of isocitrate dehydrogenase (IDH) mutant tumors indicated a superior performance of PWI to [^18^F]FET PET (AUC = 0.8/< 0.62, *p* < 0.01/≥ 0.3).

**Conclusion:**

While marked hyperperfusion on PWI indicated TP, [^18^F]FET PET proved beneficial to discriminate TP from TRC when PWI remained inconclusive. Thus, our results highlight the clinical value of sequential use of PWI and [^18^F]FET PET, allowing an economical use of diagnostic methods. The impact of an *IDH* mutation needs further investigation.

**Supplementary Information:**

The online version contains supplementary material available at 10.1007/s00259-020-05114-0.

## Introduction

Following brain cancer treatment, the early and reliable detection of tumor progression (TP) is of paramount clinical interest [1]. The imaging standard for glioma proposed by the Response Assessment in Neuro-Oncology (RANO) group is the morphological approach in magnetic resonance imaging (MRI) with diffusion-weighted sequences [2]. A limitation of this method is the sometimes insufficient differentiation of TP from solely treatment-related changes (TRC) [3]. Supplementary perfusion-weighted MRI (PWI) is widely used [4] in order to improve diagnostic accuracy [5]. Several studies demonstrated the benefit of dynamic susceptibility contrast (DSC) PWI in high-grade glioma [6–8]. As opposed to the mainly inflammatory processes of TRC [3], the neoplastic hypervascularization in glioma can result in a relative increase of the cerebral blood volume compared to normal-appearing brain tissue (rCBV) [6]. The reliability of this method, however, is controversial and for example, Boxerman et al. [7] were not able to differentiate TP on the basis of a single rCBV measurement and instead suggested a longitudinal approach. Another option for distinguishing between TP and TRC is the use of PET with radiolabeled amino acids such as O-(2-[^18^F]fluoroethyl-)-l-tyrosine ([^18^F]FET) [9, 10]. [^18^F]FET can pass through the blood-brain barrier and is taken up into the cells by amino acid transporters [10]. While the exact uptake mechanism and regulation is not fully understood [11], it has been demonstrated that the extent of [^18^F]FET uptake in most high-grade gliomas as well as in the majority of low-grade gliomas [9] is considerably higher than in normal brain tissue [1]. Also, the dynamic of the uptake differs, allowing for further distinction [11]. The same holds true when comparing the tumor uptake to that of inflammatory processes [11]. Previous studies specifically investigating the differentiation of TP and TRC in glioma [12–16] reported diagnostic accuracies of [^18^F]FET PET between 81% [17] and 99% [18]. This considerable range could be attributed to the analysis of different PET parameters and the particular patient populations, varying in tumor subtypes and treatments.

Several analyses correlated [^18^F]FET PET parameters with PWI-derived parameters like rCBV [19–22]. However, there is a general consensus that [^18^F]FET uptake, especially at 20–40 min, is dominated by the expression of amino acid transporters [10, 19], explaining why hotspot locations on [^18^F]FET PET and PWI do often not coincide [22]. Specific comparisons of the diagnostic value of [^18^F]FET PET and PWI to differentiate TP from TRC have only been performed in smaller cohorts (26–47 patients) missing molecular markers [23–25]. The results ranged from superiority of [^18^F]FET PET [23, 24] to equal performance of both methods [25] and indicated an added value of combined data.

In the present study, we retrospectively evaluated the data of dynamic [^18^F]FET PET and DSC PWI from patients with suspected recurrent glioma with a focus on the additive value of sequentially implementing both methods for the clinical decision-making process at our center. On the basis of a relatively large patient cohort, we analyzed the diagnostic accuracy of different parameters and possibly beneficial combinations thereof. Furthermore, we included a subgroup analysis of tumors with and without isocitrate dehydrogenase (IDH) mutation.

## Methods

### Patient selection

This retrospective study was approved by the scientific board of the University Cancer Center Frankfurt and the local ethics committee (SNO-8-2018). All patients had given written informed consent. PWI measurements were conducted at the Institute of Neuroradiology, Goethe University Hospital Frankfurt. [^18^F]FET PET imaging was performed at the Research Center Juelich, Germany.

We searched our database for adults with (1) histologically proven glioma who underwent both [^18^F]FET PET and PWI in order to differentiate between TP and TRC (triggered through previous MRI findings suspicious for progressive disease according to RANO) and (2) a maximum of 3 months between the two examinations without changes in treatment or neurosurgical intervention.

### Imaging protocols and post-processing

**Fig. 1:**
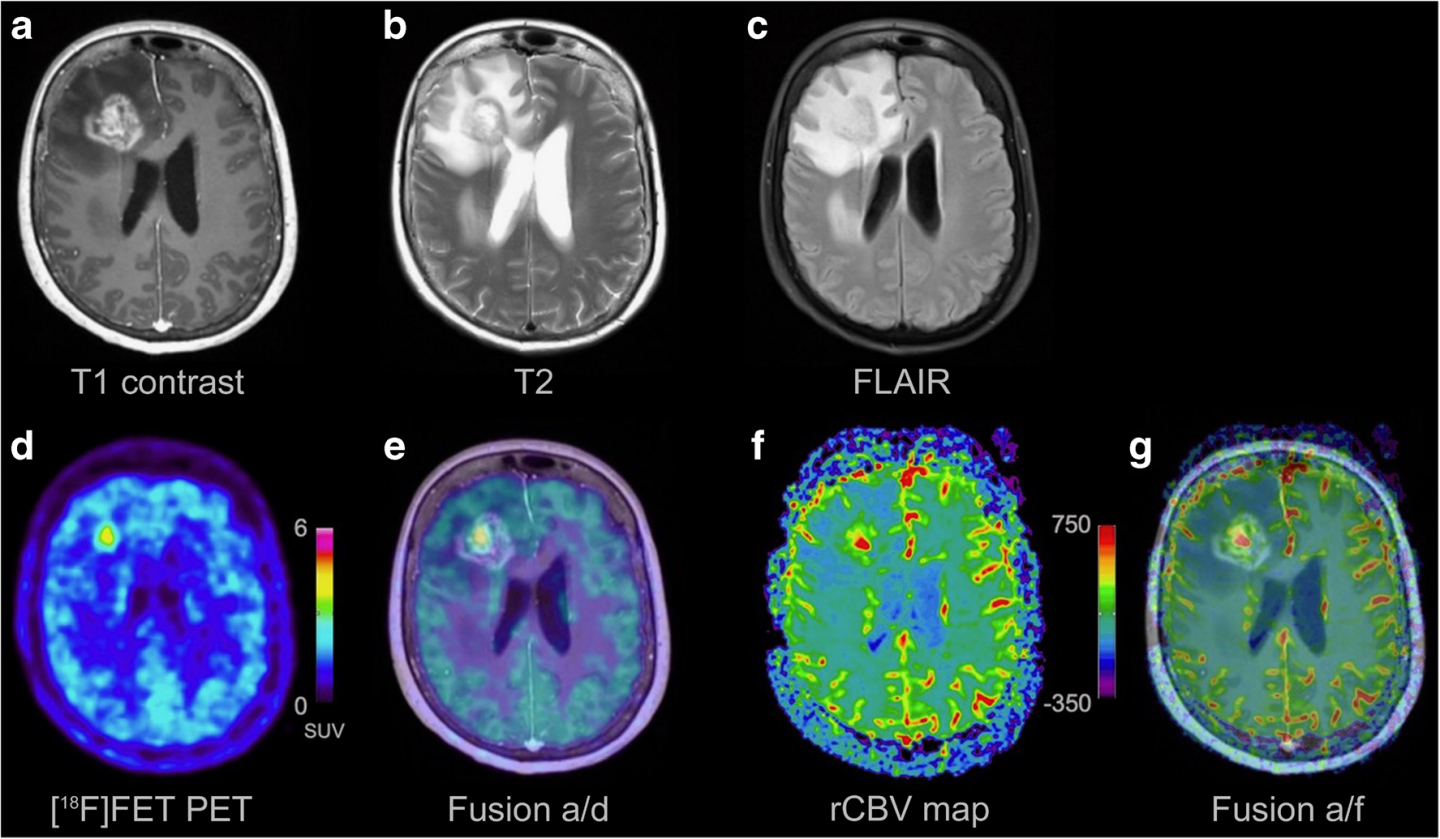
Example of corresponding PWI and [^18^F]FET PET. A T1-weighted contrast-enhanced (**a**), a T2-weighted (**b**), and a fluid-attenuated inversion recovery (FLAIR, **c**) MR image of a patient with tumor progression (TP). The corresponding [^18^F]FET PET (map only shown in **d**; map as transparent overlay on the T1-weighted contrast-enhanced image shown in **e**) and PWI-derived cerebral blood volume map (CBV; map only shown in **f**; map as transparent overlay on the T1-weighted contrast-enhanced image shown in **g**) demonstrate a hotspot in the right frontal lobe

DSC PWI measurements were performed on two MR scanners (1.5 Tesla Achieva dStream®, Philips Healthcare, Amsterdam, Netherlands; 3 Tesla Skyra®, Siemens, Erlangen, Germany). The protocols for the perfusion measurements were adapted to the respective scanner performance (1.5/3 Tesla), thus differing in detail but had not been changed over the examined time period (gradient-echo echo-planar imaging; time-to-echo, 30–38 ms; time-to-repeat, 1790–2104 ms; flip-angle, 90°; slice thickness, 3–5 mm; 50 dynamic scans). Measurements were performed both with and without application of a contrast agent prebolus before applying the intravenous main bolus (gadolinium-based agent, 0.1 mmol/kg bodyweight; infusion rate, 4 ml/s followed by 21 ml of NaCl). Corresponding anatomical MRI including T2- and contrast-enhanced T1-weighted images was available. All raw data were reanalyzed for this study. We used the automated MR Neuro Perfusion application within the Philips IntelliSpace® software toolbox. Post-processing leakage correction based on the Boxerman-Weisskoff approach was employed [26]. The calculated perfusion maps were co-registered and used as an overlay on anatomical MRI to allow for vessel exclusion and identification of tumor margins. The area of maximum CBV within the tumor was then visually assessed and mapped as ROI. An equally sized ROI in the contralateral, normal-appearing brain tissue was used for calculation of the maximum rCBV (rCBV_max_ = CBV_tumor_/CBV_normal tissue_). Figure 1 shows exemplary images from PWI and [^18^F]FET PET analysis. The ROI selection was conducted in consensus by two radiologists (E.H. and E.S.) who were blinded to both diagnosis (including [^18^F]FET PET data) and outcome of the patients. To assess the inter-rater reliability, measurements were reanalyzed by another experienced neuroradiologist (F.K.), who was previously not engaged in the project and also blinded.

Detailed information on [^18^F]FET PET acquisition (stand-alone PET scanner ECAT EXACT HR+ and 3-T hybrid PET/MR scanner BrainPET; both Siemens Healthcare, Erlangen, Germany) and post-processing was published recently [17]. Parameters evaluated were the region of interest (ROI) based mean and maximum tumor to brain ratios (TBR_mean_, TBR_max_), the time-to-peak of the time-activity curve in minutes (TTP), and the slope of the time-activity curve 20–40 min post-injection expressed in change of standardized uptake value per hour (SUV/h, Slope; SUV = image activity concentration [Bq/g] * patient weight [g] ∕ injected activity [Bq]). All analyses were conducted in the clinical context while the final diagnosis and the PWI results were still unknown.

### Final diagnosis of TP and TRC

The final diagnosis was based either on histopathology as previously published [17] or clinico-radiological follow-up as specified below.

TRC was diagnosed if the following criteria applied: For WHO grade II gliomas, the clinical and radiological assessment had to be stable or improved for a minimum of 12 months without the administration of another therapy. For WHO grade III–IV gliomas, at least 6 months of stable or improved clinical and radiological status, as well as unchanged treatment were necessary.

Congruently, TP was diagnosed if the following criteria applied: A continued growth of target lesions over at least 6 months (rated as progressive disease according to RANO) and at least two subsequent MRI scans as well as a paralleled deterioration in performance status were required. The diagnosis of tumor progression on a single MRI according to RANO criteria with subsequent tumor-related death preventing further examinations was also adequate.

### Statistics

Intergroup differences were assessed with the Mann-Whitney *U* test (SPSS Statistics 26®, IBM, New York, USA). The diagnostic performances for the differentiation between TP and TRC were evaluated by the receiver operating characteristic (ROC) procedure using the final diagnosis as reference. Cutoffs were considered optimal at the maximum of the product of sensitivity and specificity. Additionally, we verified our classification model by calculating a leave-one-out cross-validation (R version 4.0.2). Correlations were assessed by Pearson’s coefficient *r*. Inter-rater reliability analysis was conducted by Cohen’s Kappa (κ). *p* < 0.05 was considered significant.

## Results

### Patients and final diagnosis

One hundred and four patients met the inclusion criteria; 84 of them had been included in a previous analysis concerning the diagnostic performance of [^18^F]FET PET [17]. They had a median age of 52 years (range 20–78 years), 34.6% of them were female (detailed characteristics in Table 1 and Supplemental Table 1). The median interval between [^18^F]FET PET and PWI was 11.5 days, with PWI being acquired first in 61% of all cases. 18 patients (17%), 11 of whom suffered from *IDH*-wild-type tumors, underwent both examinations with a lag of more than 30 days. Four out of those 11 patients were correctly diagnosed with TP by the second examination but not by the first one ([^18^F]FET PET and PWI in two individuals each), suggesting that, in these instances, the relatively long interval could have biased the assessment of diagnostic accuracy. All examinations took place between February 2016 and December 2019. The final diagnosis of TP (*n* = 83) and TRC (*n* = 21) was based on histopathology in 42 cases (40%; resection, *n* = 35 (including 5 TRC cases); biopsy, *n* = 7 (including 1 TRC case)) and on follow-up in 62 cases. Subgroups with histology (14% TRC) or follow-up-based diagnosis (24% TRC) displayed no significant differences for all evaluated imaging parameters (*p* > 0.1). ROC analysis for the identification of TP yielded identical areas under the curve (AUC) for rCBV_max_ in both subgroups (AUC histology, 0.755; *p* = 0.048; AUC follow-up, 0.756; *p* < 0.01) (Supplemental Figure 1).

**Table 1:** Tumor characteristics (all patients, *n* = 104)

**Diagnosis (WHO 2016)**	
Oligodendroglioma, *IDH*-mutant and 1p/19q-codeleted	10 (9.6 %)
WHO grade II	3
WHO grade III	7
Astrocytoma, *IDH*-mutant	15 (14.4 %)
WHO grade II	3
WHO grade III	12
Astrocytoma, *IDH*-wild type	6 (5.8 %)
WHO grade II	2
WHO grade III	4
Astrocytoma, NOS, WHO grade III	1 (1 %)
Diffuse glioma, NOS, WHO grade II	1 (1 %)
Diffuse midline glioma, H3K27M-mutant, WHO grade IV	1 (1 %)
Glioblastoma, *IDH*-mutant, WHO grade IV	8 (7.7 %)
Glioblastoma, *IDH*-wild type, WHO grade IV	61 (58.7 %)
Glioblastoma, NOS, WHO grade IV	1 (1 %)
**Molecular markers**	
*IDH*-status	
Mutant	33 (31.7 %)
Wild type	69 (66.3 %)
Not available/ inconclusive	2 (1.9 %)
*MGMT*-promoter status	
Methylated	52 (50 %)
Unmethylated	35 (33.7 %)
Not available/inconclusive	17 (16.3 %)
**Therapy**	
Radiotherapy	102 (98.1 %)
Re-irradiation	17 (16.3 %)
Chemotherapy	98 (94.2 %)
Temozolomide	95 (91.3 %)
Lomustine	32 (30.8 %)
Bevacizumab	8 (7.7 %)
Nivolumab	6 (5.8 %)
Tumor-treating fields	10 (9.6 %)
Re-resection	21 (20.2 %)
Interval between last therapy and[^18^F]FET PET scan, days, median (range)	58 (0–2963)

*IDH*, isocitrate dehydrogenase; *NOS*, not otherwise specified; *MGMT*, O^6^-methylguanine-DNA methyl-transferase

### TP versus TRC

**Fig. 2:**
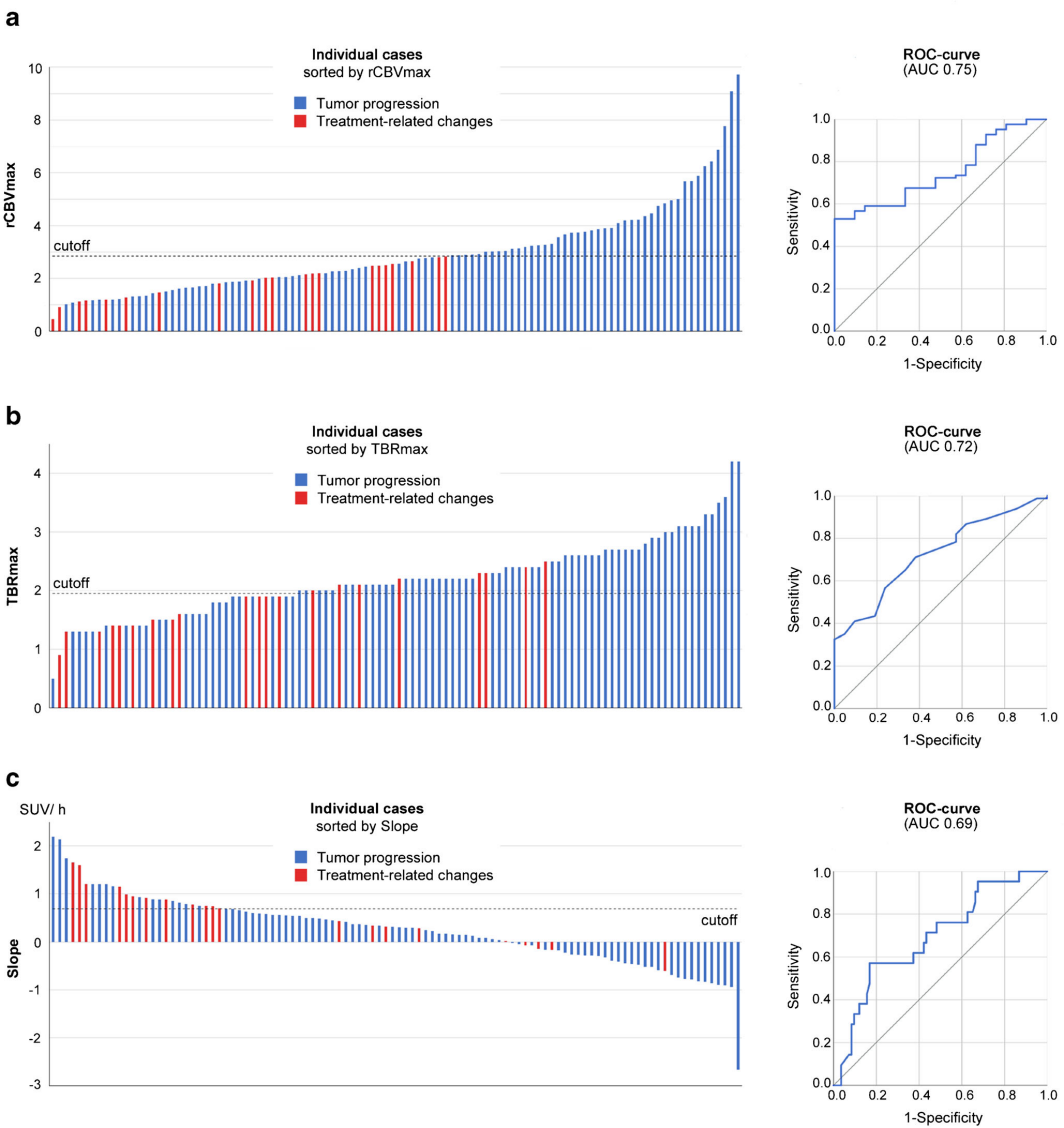
Case distribution and ROC curves. Cases were sorted by either the maximum relative cerebral blood volume (rCBV_max_, **a**), the maximum tumor to brain ratio (TBR_max_, **b**), or the Slope (**c**), and corresponding receiver operating characteristic (ROC) curves are depicted. The dotted lines indicate the optimal cutoff as determined by the maximum product of sensitivity and specificity. AUC, area under the curve; SUV/h, standardized uptake value per hour

Values for TBR_mean_, TBR_max_, and rCBV_max_ were significantly higher in TP than in TRC and significantly lower for Slope. TTP values did not differ (Table 2). For rCBV_max_, the difference remained significant within the subgroup of patients with histologically proven diagnosis (*p* = 0.048, *n* = 42). The inter-rater reliability for rCBV_max_ was *κ* = 0.81. ROC analysis for detecting TP yielded significant results for all parameters but TTP (Table 2, Fig. 2). For further evaluation, we excluded TTP for missing significance and TBR_mean_ for redundancy to TBR_max_ (*r* = 0.93). Optimal cutoffs to identify TP (TBR_max_, Slope, rCBV_max_), as well as the resulting sensitivities, specificities, accuracies, positive predictive (PPV), and negative predictive values (NPV), are given in Table 2.

**Table 2:** TP versus TRC

	TP (MAD)	TRC (MAD)	p*	AUC (95% CI)	*p*^†^	Cutoff	Sens (95% CI)	Spec (95% CI)	Acc (95% CI)	PPV (95% CI)	NPV (95% CI)
rCBV_max_	2.90 (1.00)	2.03 (0.52)	< 0.00	0.75 (0.65–0.85)	< 0.00	> 2.85	0.54 (0.42–0.64)	1.0 (0.84–1)	0.63 (0.52–0.72)	1.0 (0.92–1)	0.36 (0.23–0.48)
TBR_max_	2.20 (0.40)	1.90 (0.40)	< 0.00	0.72 (0.61–0.83)	< 0.00	> 1.95	0.70 (0.59–0.79)	0.60 (0.38–0.82)	0.68 (0.58–0.77)	0.88 (0.78–0.95)	0.32 (0.20–0.51)
Slope^‡^	0.23^‡^ (0.45)	0.74^‡^ (0.41)	< 0.01	0.69 (0.57–0.82)	< 0.01	< 0.69^‡^	0.84 (0.73–0.90)	0.62 (0.34–0.78)	0.80 (0.69–0.85)	0.90 (0.79–0.95)	0.50 (0.27–0.67)
TBR_mean_	2.00 (0.20)	1.90 (0.20)	< 0.00	0.72 (0.61–0.83)	< 0.00						
TTP^§^	32.5^§^ (5.00)	32.5^§^ (5.00)	0.14	0.60 (0.50–0.72)	0.16						
TBR_max_ a/o Slope^║^	n.a.	n.a.	n.a.				0.96 (0.90–0.99)	0.43 (0.22–0.66)	0.86 (0.77–0.92)	0.87 (0.78–0.93)	0.75 (0.43–0.95)

*TP*, tumor progression (median value); *TRC*, treatment-related changes (median value); *MAD*, median absolute deviation; *AUC*, area under the curve; *95% CI*, 95% confidence interval; *Sens*, sensitivity; *Spec*, specificity; *Acc*, accuracy; *PPV*, positive predictive value; *NPV*, negative predictive value; *rCBV*_*max*_, maximum relative cerebral blood volume; *TBR*_*max*_, maximum tumor to brain ratio; *TBR*_*mean*_, mean tumor to brain ratio; *TTP*, time-to-peak; *a/o*, and/or

*p values for the intergroup comparison of TP and TRC (Mann-Whitney *U* test)

^†^*p* values for the AUC

^‡^In standardized uptake value per hour (SUV/h)

^§^In minutes

^**║**^TP is assumed if any of the values crosses the cutoff specified for the individual parameter

All ratios (rCBV_max_, TBR_max_, TBR_mean_) were calculated by dividing the value measured in tumor through the value measured in contralateral, normal-appearing brain tissue

When considering only *IDH*-wild-type tumors (*n* = 69), ROC curves for TBR_max_ and Slope slightly improved (AUC, 0.79, 95% confidence interval (95% CI) 0.67–0.92, and 0.77, 95% CI 0.62–0.92; *p* < 0.00), while the AUC for rCBV_max_ slightly decreased (AUC, 0.72, 95% CI 0.59–0.85; *p* = 0.02). An opposing effect was present in *IDH*-mutant gliomas (*n* = 33). Particularly, the parameter Slope lost significance (AUC Slope, 0.48, 95% CI 0.30–0.74; *p* = 0.85; AUC TBR_max_, 0.62, 95% CI 0.41–0.82; *p* = 0.3) while the performance of rCBV_max_ increased (AUC, 0.8, 95% CI 0.65–0.95; *p* < 0.01, Supplemental Figures 2 and 3).

### Sequential application of PWI and [^18^F]FET PET

**Fig. 3:**
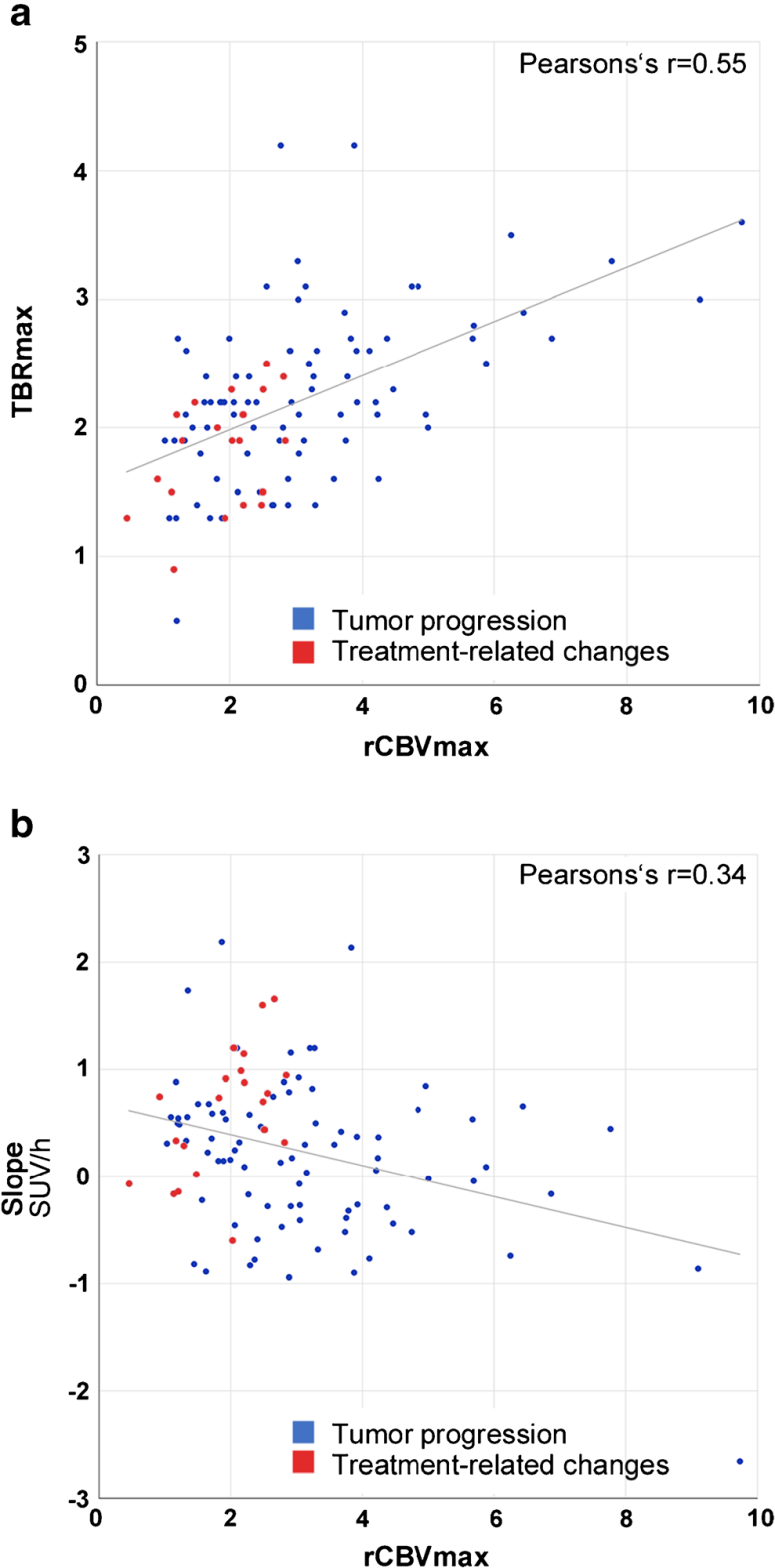
Correlation of PWI and [^18^F]FET PET parameters. Data of the maximum relative cerebral blood volume (rCBV_max_) and the maximum tumor to brain ratio (TBR_max_) (**a**) and of the rCBV_max_ and the Slope (**b**) are displayed in scatter plots with a regression line. Dots colored in red represent cases with a final diagnosis of treatment-related changes; blue dots represent cases with tumor progression. SUV/h, standardized uptake value per hour

**Fig. 4:**
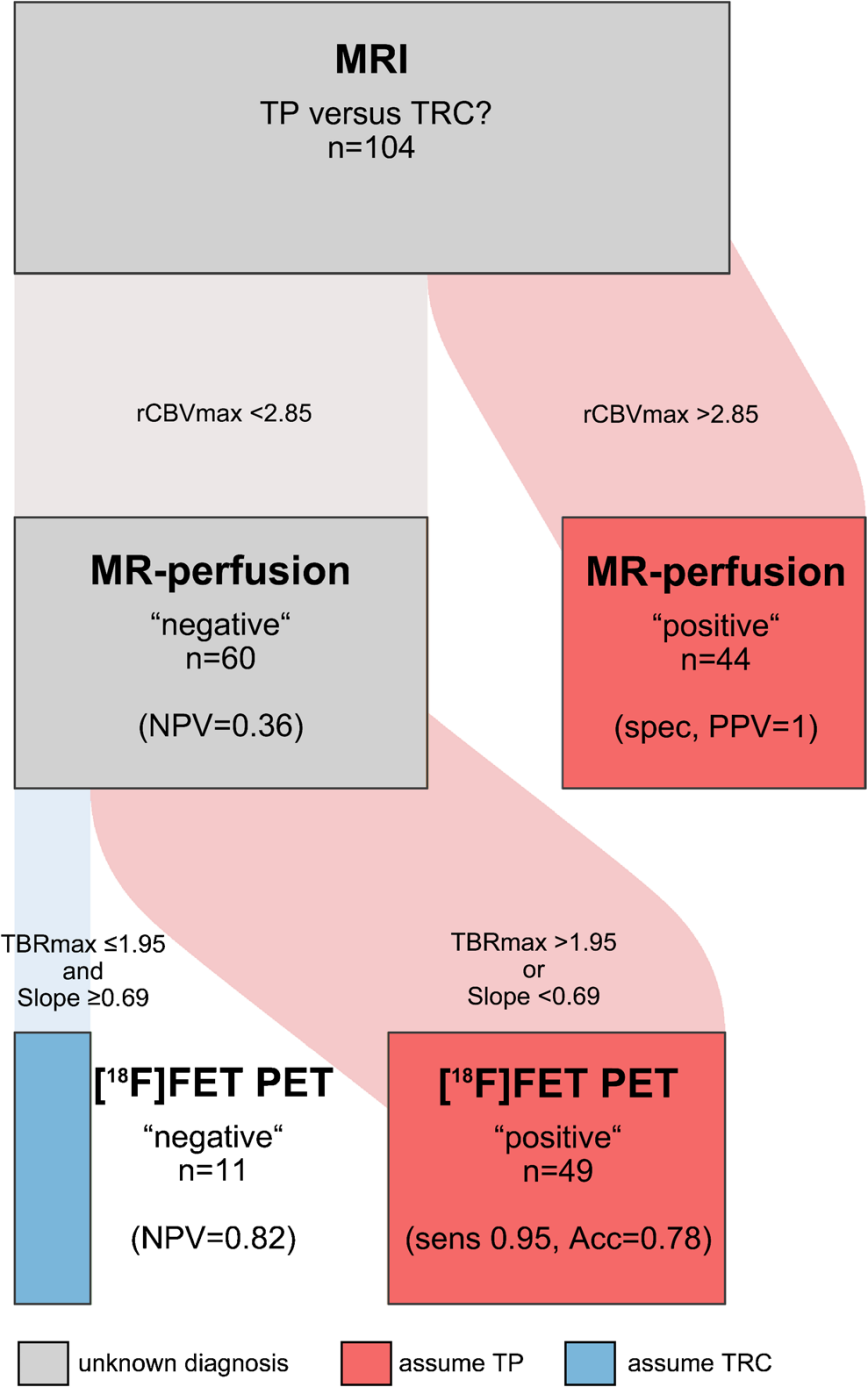
Flow chart for the sequential use of PWI and [^18^F]FET PET. The width of the boxes and the connecting flows are proportional to the number of patients. The complete cohort is depicted by the gray box at the top (*n* = 104). Assuming tumor progression (TP) if the maximum relative cerebral blood volume (rCBV_max_) is above 2.85 classifies 44 patients (red box, middle right) and leaves 60 patients unclassified (gray box, middle left). Further classification as TP (red box, bottom right, *n* = 49) is conducted if either the maximum tumor to brain ratio (TBR_max_) is above 1.95 or the Slope is below 0.69 SUV/h (standardized uptake value per hour). Treatment-related changes (TRC) are assumed if both parameters do not cross the cutoff (blue box, bottom left, *n* = 11). Acc, accuracy; NPV, negative predictive value; PPV, positive predictive value; sens, sensitivity; spec, specificity

There was an intermediate correlation between rCBV_max_ and TBR_max_ (*r* = 0.55) and no correlation between both rCBV_max_ and TBR_max_ and Slope (*r* = 0.34 and 0.32; Fig. 3 and Supplemental Figure 4). In a sequential approach (Fig. 4), all cases with a rCBV_max_ value above the cutoff of 2.85 (*n* = 44) were correctly classified as TP (specificity, 1.0; PPV 1.0). In the remaining 60 cases (21 with TRC), PWI was insufficient for a diagnostic classification. By contrast, especially the [^18^F]FET PET parameter Slope remained significant in ROC analysis (AUC Slope, 0.66; *p* = 0.04; AUC TBR_max_, 0.61; *p* = 0.18) and the combination of Slope and TBR_max_ (assuming TP if either value crossed the cutoff) achieved an accuracy of 78% (sensitivity, 0.95; specificity, 0.45; PPV, 0.78; NPV, 0.82). Overall, combined PWI and [^18^F]FET PET reached an accuracy of 87% (sensitivity 98%; specificity 43%). Performing a leave-one-out cross-validation for this classification approach resulted in a comparable accuracy of 83% (sensitivity 96%; specificity 25%).

## Discussion

Our study addressed the diagnostic value of sequential DSC PWI and dynamic [^18^F]FET PET to differentiate TP from TRC.

The [^18^F]FET PET parameters TBR_max/mean_ and Slope, as well as the MR-derived rCBV_max_, yielded a moderate diagnostic performance to discriminate between TP and TRC (AUC, 0.69–0.75; Fig. 2). Since the AUC values for these parameters were comparable, our findings did not confirm previous observations in smaller cohorts that reported an inferiority of PWI to [^18^F]FET PET [23, 24]. Noteworthy and in line with previous reports [23, 24], the sensitivity of the rCBV_max_ was rather low (0.53), while the sensitivity of the combined TBR_max_ and slope values was substantially higher (0.96). A likely explanation for the low sensitivity of rCBV_max_ [6, 27] is the fact that even at initial diagnosis glioma of all grades can lack increased perfusion [6, 28–31]. Consequently, “negative” PWI results do not reliably indicate TRC.

In contrast to the low sensitivity, the high rCBV_max_ cutoff [6] as determined by ROC analysis yielded a high specificity for rCBV_max_. As depicted in Fig. 2, the rCBV_max_ for TRC did not exceed 2.85. According to the literature, this is likely true for most cases and explains the specificity of DSC PWI [6, 27].

Based on the high specificity of PWI on the one hand and the high sensitivity of [^18^F]FET PET on the other hand, we analyzed the additive diagnostic value of a sequential combination of both examinations (Fig. 4). Correlating PWI and [^18^F]FET PET parameters revealed a closer relationship between the static parameters rCBV_max_ and TBR_max_ than between the static parameters rCBV_max_ and TBR_max_ and the dynamic parameter Slope. Göttler et al. [21] described similar findings when analyzing voxel-wise correlations, indicating that the maximum [^18^F]FET uptake might depend more on high blood volumes than the washout parameter Slope.

In the first step, cases above the rCBV_max_ cutoff were classified as TP. This allowed a correct classification of 42% of all patients and necessitated further evaluation by [^18^F]FET PET in the remaining 58%. Importantly, a significant discrimination of TP and TRC by [^18^F]FET PET was still possible in this preselected subgroup. The combination of Slope and TBR_max_ achieved an accuracy of 78% and correctly classified another 45% of all patients. Altogether, this stepwise strategy led to a correct diagnosis in 87% of the 104 patients with a good stability in the cross-validation. Thus, in the majority of patients, the sequential combination of PWI and [^18^F]FET PET allowed for a reliable differentiation of TP and TRC in this crucial diagnostic situation. In a significant proportion of patients, it could also help to avoid the additional effort and cost of [^18^F]FET PET.

Our previous study on the diagnostic performance of [^18^F]FET PET with a partially overlapping cohort revealed a lower performance of [^18^F]FET PET in *IDH*-mutant than in *IDH*-wild-type tumors [17]. In contrast to the observations with [^18^F]FET PET, the AUC value for rCBV_max_ even increased in the subgroup of *IDH*-mutant tumors. According to the literature, the perfusion properties of *IDH*-mutant gliomas are controversial. Results range from lower [28] to equal [29, 30] to higher rCBV values [31] in comparison to *IDH*-wild-type tumors and are at least to some extent influenced by the selection of cohorts according to WHO grade. Due to the lack of comparable studies and the small number of 33 patients, further research to validate and elucidate our finding is necessary before implementation into a diagnostic algorithm can be discussed. As our cohort only included 10 tumors harboring a 1p/19q co-deletion, we refrained from the further genetic subgroup analysis.

### Limitations

As we did not limit our study to high-grade gliomas or specific treatment regimens [15, 16, 32–36], our patient cohort is inhomogeneous. Besides, it is likely biased towards difficult cases, because only patients with ambiguous MRI findings and remaining therapeutic options were referred to [^18^F]FET PET imaging. The final diagnosis was based on histology in 40% of our patients, which is an average rate [27]. Especially the decision to perform a resection might have been biased by the suspicion of actual tumor progression. The comparatively high patient number, however, allowed a comparison of the subgroups with histology and follow-up-based diagnosis. The absence of any significant differences between these subgroups can be regarded as a verification of our follow-up criteria to some extent. As opposed to other combined PET-MRI studies, our examinations were not conducted simultaneously, but the median interval between the examinations of 11.5 days was reasonable and reflects the current procedure for most patients. Some aspects of our PWI analysis are limited by the utilization of different MR scanners and protocols. Yet, reporting individually normalized, relative values, homogeneously reanalyzing all data, and employing a leakage correction presumably minimized the ensuing inaccuracy [37]. Nevertheless, for future studies, the new consensus PWI protocol [38] should be implemented to promote reproducibility and the exact rCBV_max_ cutoffs reported in this study remain somewhat specific to this dataset. Lastly, the presented data are solely based on reproducible, quantitative parameters. For both [^18^F]FET PET and MRI, the actual clinical assessment may be more accurate when other factors such as the morphologic appearance of the imaging changes in question and the tracer distribution are considered by an experienced radiologist or nuclear medicine physician.

## Conclusion

Our results favor a combined and sequential use of PWI and [^18^F]FET PET for the differentiation of TP and TRC in gliomas, providing reliable results in the majority of patients. Abnormal PWI permitted a definite diagnosis of TP in 42% of the patients, and subsequent [^18^F]FET PET allowed a correct classification in another 45%. We propose this stepwise approach as a resource-sparing and cost-effective strategy, when a categorization is necessary to facilitate clinical decision-making. In the subgroup of *IDH*-mutant tumors, PWI appeared to be more reliable than [^18^F]FET PET, which is a surprising finding and needs further validation.

## Supplementary information

Supplemental fig. 1 ROC curves in subgroups with histology or follow-up-based diagnosis Receiver operating characteristic (ROC) curves for maximum relative cerebral blood volume (rCBV_max_) in patients with final diagnosis established by (a) clinico-radiological follow-up (n=62) and (b) histopathology (n=42), respectively. AUC, area under the curve (EPS 11336 kb)

High Resolution Image (PNG 92 kb)

Supplemental fig. 2 ROC curves in *IDH*-mutant tumors In the subgroup of *IDH*-mutant gliomas, receiver operating characteristic (ROC) curves for maximum relative cerebral blood volume (rCBV_max_, red), maximum tumor-brain-ratio (TBR_max_, blue) and Slope (black) are plotted in one graph. AUC, area under the curve; IDH, isocitrate dehydrogenase (EPS 13337 kb)

High Resolution Image (PNG 110 kb)

Supplemental fig. 3. Case distribution for *IDH*-mutant tumors Cases were sorted by either the maximum relative cerebral blood volume (rCBV_max_, A), the maximum tumor-brain ratio (TBR_max_, b) or the Slope (c). The dotted lines indicate the optimal cutoff as determined by the maximum product of sensitivity and specificity for the whole cohort. SUV/h, standardized uptake value per hour (EPS 7761 kb)

High Resolution Image (PNG 76 kb)

Supplemental fig. 4 Correlation of TBR_max_ and Slope Data of the maximum tumor-brain ratio (TBR_max_) and the Slope are displayed in a scatter plot with a regression line. SUV/h, standardized uptake value per hour (EPS 19062 kb)

High Resolution Image (PNG 51 kb)

ESM 5 (XLSX 19 kb)

## Data Availability

All data are available in the manuscript/supplementary material.
