# Supplementary figures and images for: Sequential implementation of DSC-MR perfusion and dynamic [18F]FET PET allows efficient differentiation of glioma progression from treatment-related changes
Source: Eur J Nucl Med Mol Imaging. 2020 Nov 26;48(6):1956–65. doi: 10.1007/s00259-020-05114-0 (PMC8113145; doi:10.1007/s00259-020-05114-0)

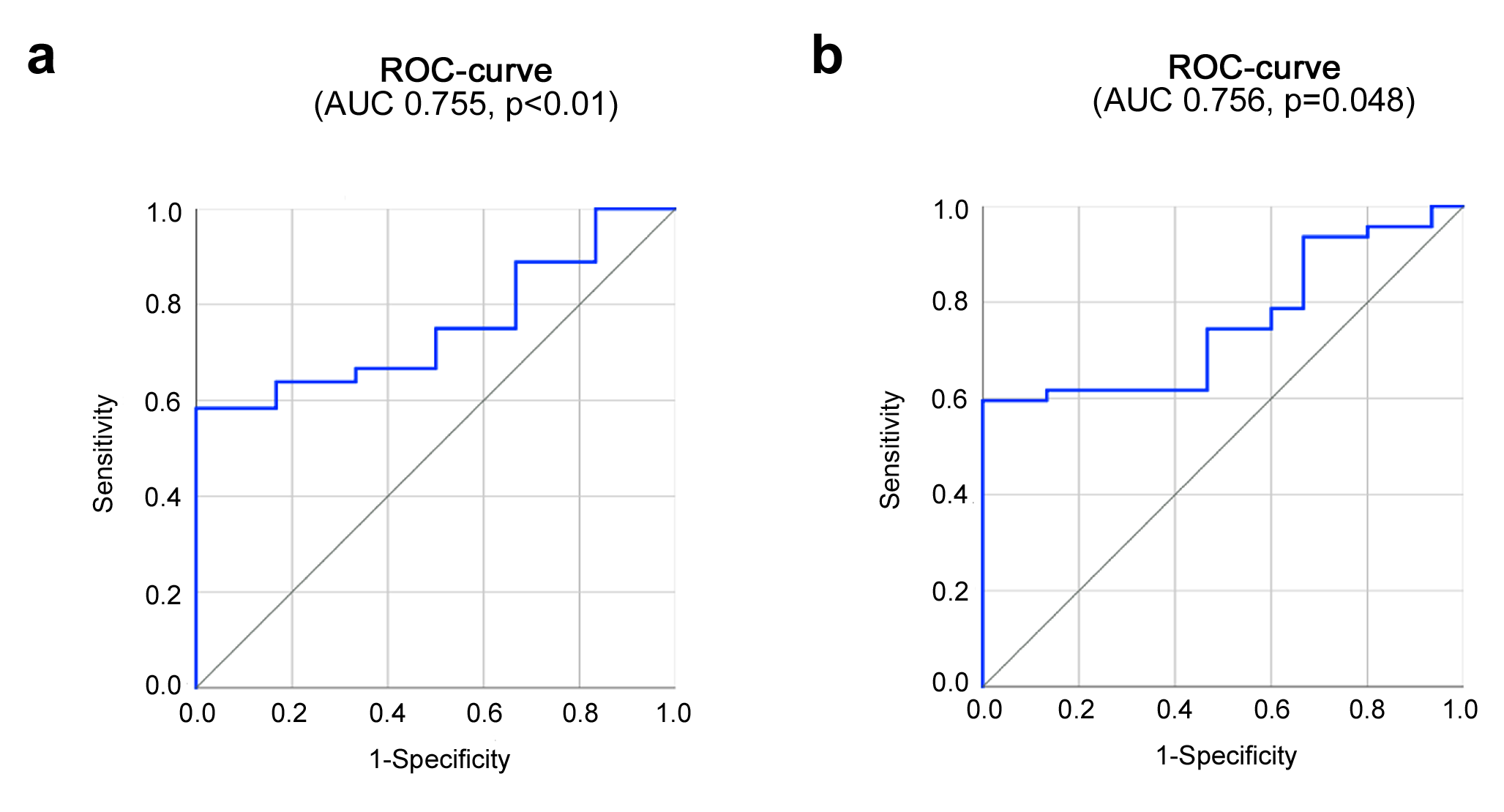

Supplement: Supplementary file 2 — High Resolution Image (PNG 92 kb) [file 259_2020_5114_FIG5_ESM.png]

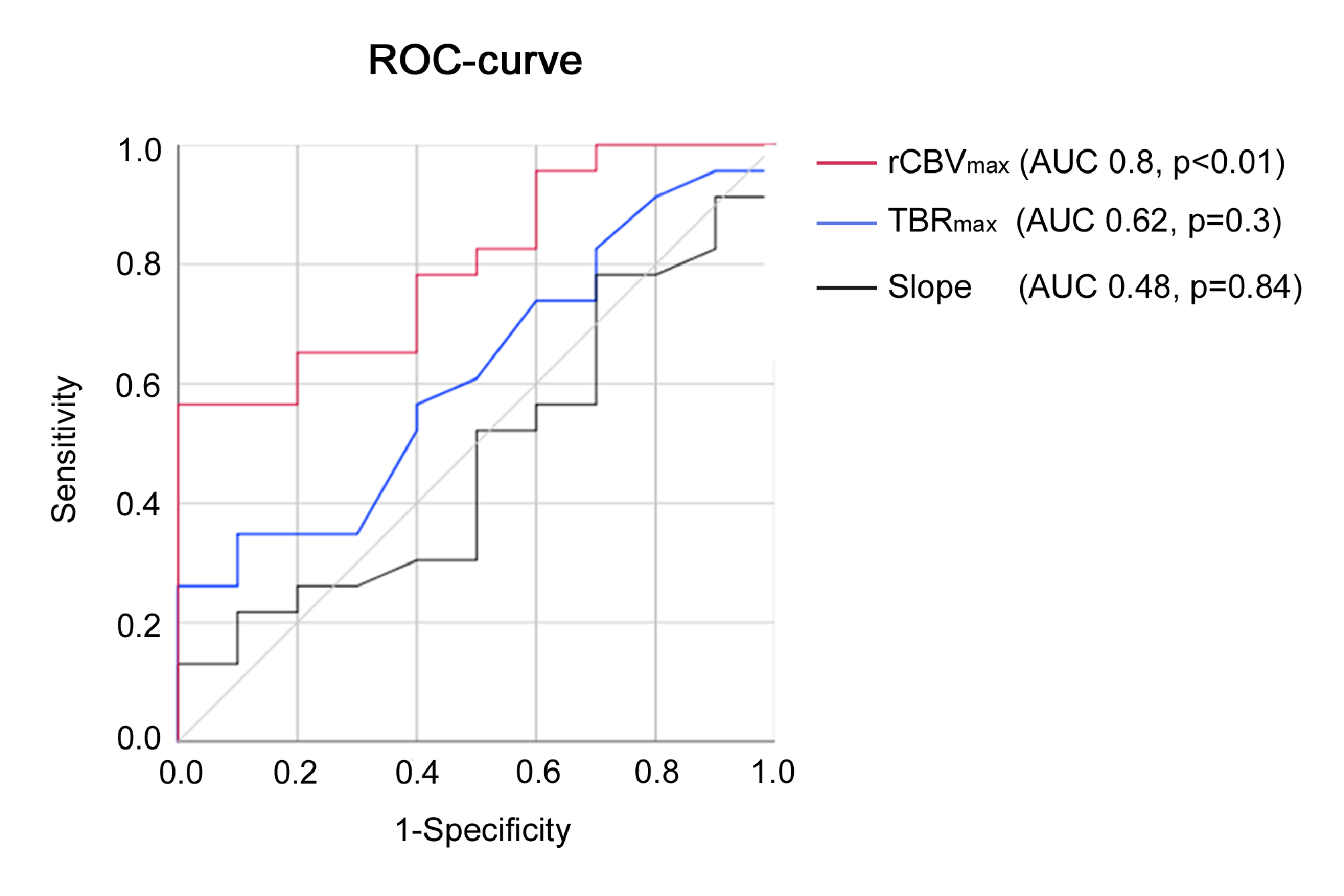

Supplement: Supplementary file 4 — High Resolution Image (PNG 110 kb) [file 259_2020_5114_FIG6_ESM.png]

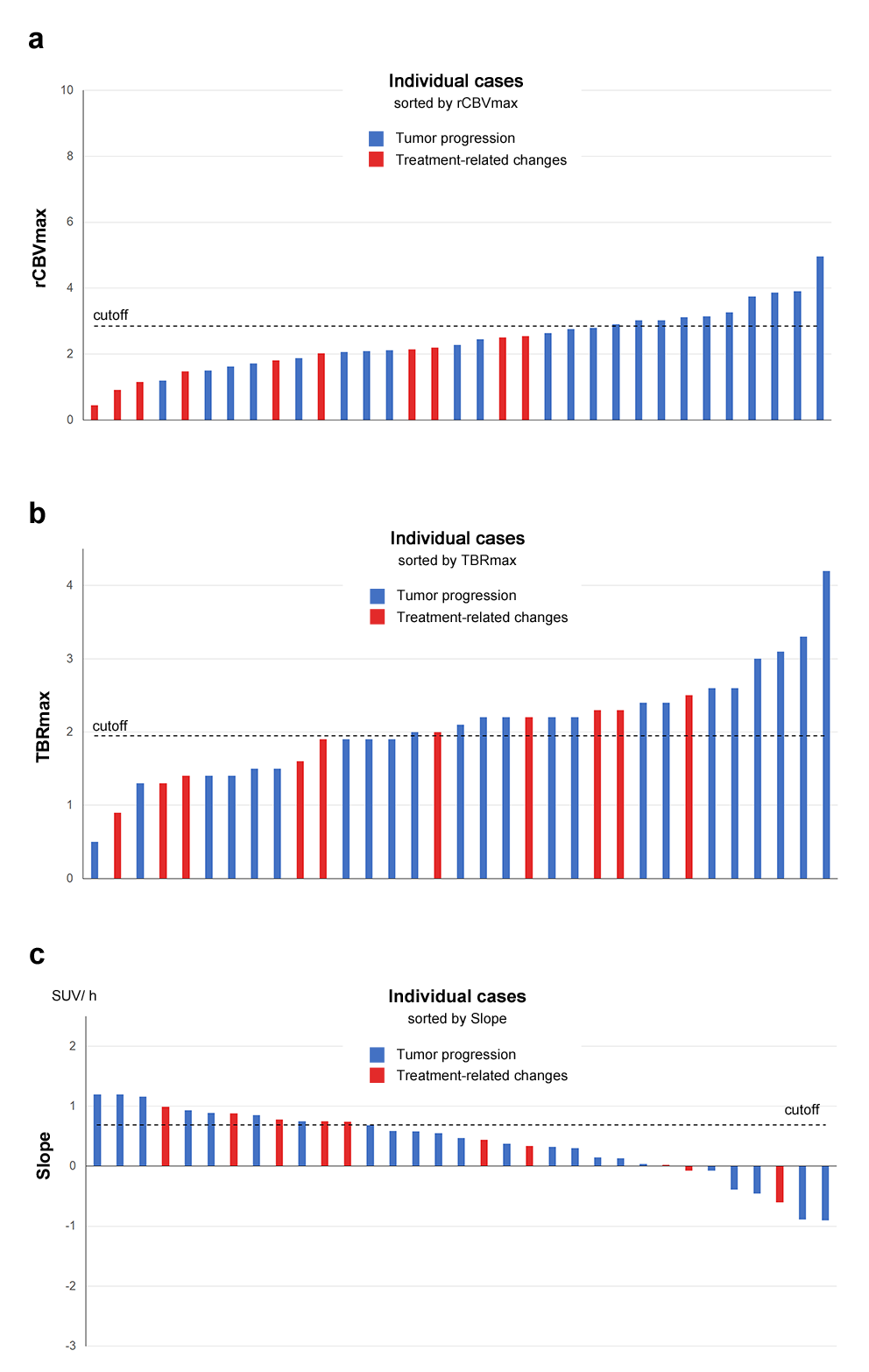

Supplement: Supplementary file 6 — High Resolution Image (PNG 76 kb) [file 259_2020_5114_FIG7_ESM.png]

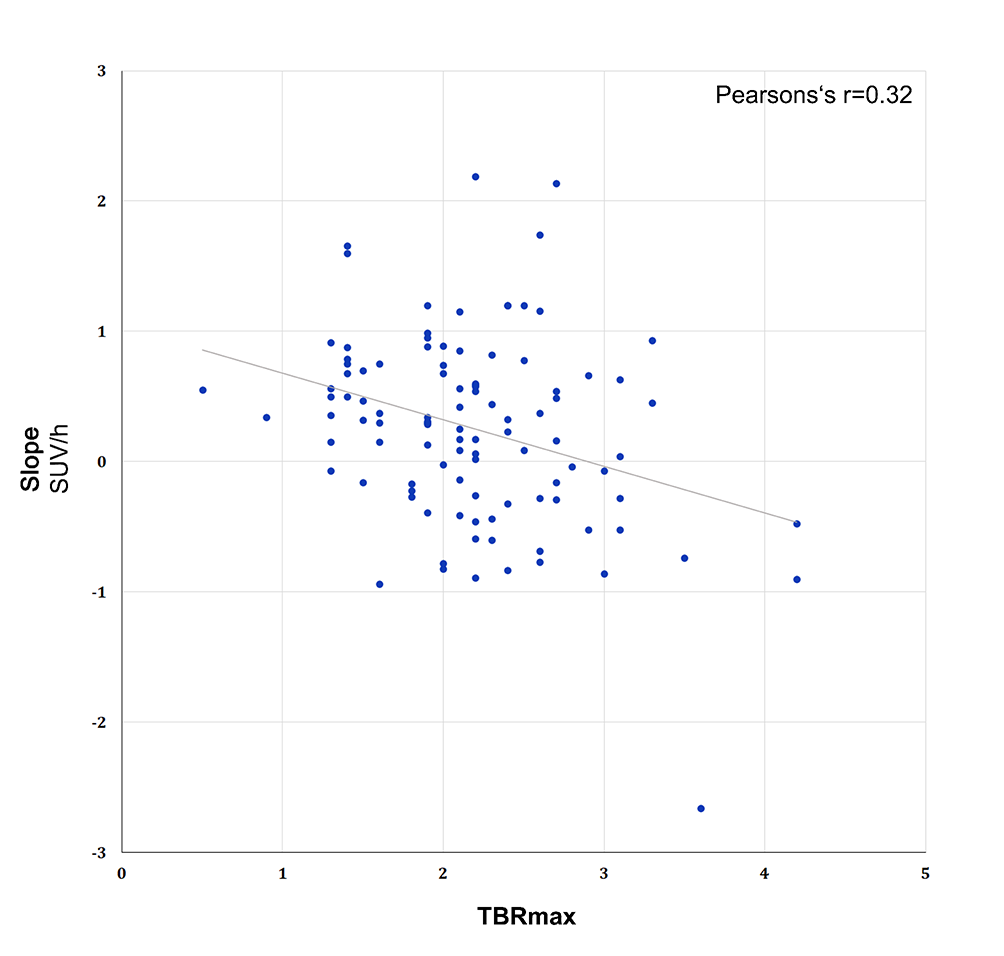

Supplement: Supplementary file 8 — High Resolution Image (PNG 51 kb) [file 259_2020_5114_FIG8_ESM.png]
